# Why Do Cryptic Species Tend Not to Co-Occur? A Case Study on Two Cryptic Pairs of Butterflies

**DOI:** 10.1371/journal.pone.0117802

**Published:** 2015-02-18

**Authors:** Raluca Vodă, Leonardo Dapporto, Vlad Dincă, Roger Vila

**Affiliations:** 1 Butterfly Diversity and Evolution Lab, Institut de Biologia Evolutiva (CSIC-Universitat Pompeu Fabra), Barcelona, Spain; 2 Departament de Genètica i Microbiologia, Universitat Autònoma de Barcelona, Bellaterra, Spain; 3 Department of Biological and Medical Sciences, Oxford Brookes University, Headington, Oxford, United Kingdom; 4 Biodiversity Institute of Ontario, University of Guelph, Guelph, Ontario, Canada; Institut National de la Recherche Agronomique (INRA), FRANCE

## Abstract

As cryptic diversity is being discovered, mostly thanks to advances in molecular techniques, it is becoming evident that many of these taxa display parapatric distributions in mainland and that they rarely coexist on islands. Genetic landscapes, haplotype networks and ecological niche modeling analyses were performed for two pairs of non-sister cryptic butterfly species, *Aricia agestis*-A. *cramera* and *Polyommatus icarus*—*P. celina* (Lycaenidae), to specifically assess non-coexistence on western Mediterranean islands, and to test potential causes producing such chequered distribution patterns. We show that the morphologically and ecologically equivalent pairs of species do not coexist on any of the studied islands, although nearly all islands are colonized by one of them. According to our models, the cryptic pairs displayed marked climatic preferences and ‘precipitation during the driest quarter’ was recovered as the most important climatic determinant. However, neither dispersal capacity, nor climatic or ecological factors fully explain the observed distributions across particular sea straits, and the existence of species interactions resulting in mutual exclusion is suggested as a necessary hypothesis. Given that the studied species are habitat generalists, feeding on virtually unlimited resources, we propose that reproductive interference, together with climatic preferences, sustain density-dependent mechanisms like “founder takes all” and impede coexistence on islands. Chequered distributions among cryptic taxa, both sister and non-sister, are common in butterflies, suggesting that the phenomenon revealed here could be important in determining biodiversity patterns.

## Introduction

Adaptation to different abiotic elements (e.g. climate, geomorphology and soil) and biotic resources determines the fundamental niche of a species. The occupancy of this theoretically suitable space is in turn constrained by other abiotic and biotic factors (such as geographic barriers and species interactions) that shape the realized niche of a species [1–4]. In the last decade, considerable progress has been made to provide researchers with essential data (climatic, environmental and distributional, e.g. GBIF http://www.gbif.org/, BIOCLIM http://www.worldclim.org/bioclim [5], Socioeconomic Data And Applications Center (SEDAC) http://sedac.ciesin.columbia.edu/) and methodological tools to unravel the relative importance of biotic and abiotic factors in determining the observed distribution of species [6–9]. Among all concurrent effects, a direct evaluation of biotic interactions such as competition and reproductive interference requires particularly complex and long-term studies [10–13]. For this reason, in most cases, the importance of species interactions is indirectly evaluated through comparative studies or by testing the power of models that, in addition to climatic and environmental variables, include biotic variables potentially correlated with the supposed interaction, such as the presence of the presumably competing species [9,14–18].

A recent study on passerine birds examined a series of sister species and revealed that rates of secondary sympatry are positively associated with both phylogenetic and morphological distance between species. The authors suggested that competition between ecologically similar species limits their possibilities to occur in sympatry [17]. The use of a large number of sister species allowed a comprehensive comparative analysis, but eliminated the possibility to examine the nature of the interactions between non-sister species that can also be morphologically and ecologically similar, despite being phylogenetically relatively distant. A recent comparative study of the entire butterfly fauna of the western Mediterranean revealed that complexes of cryptic taxa show lower co-occurrence than other congeneric non-cryptic species [19]. Among these, several examples of non-sister cryptic taxa with chequered distributions have been reported. Interestingly, such ecologically and morphologically similar species tend to be parapatric on mainland and apparently many display chequered distributions on islands, even over narrow sea straits [20–25]. Such a pattern could represent a signal of interspecific interactions [17,26,27].

**Fig 1:**
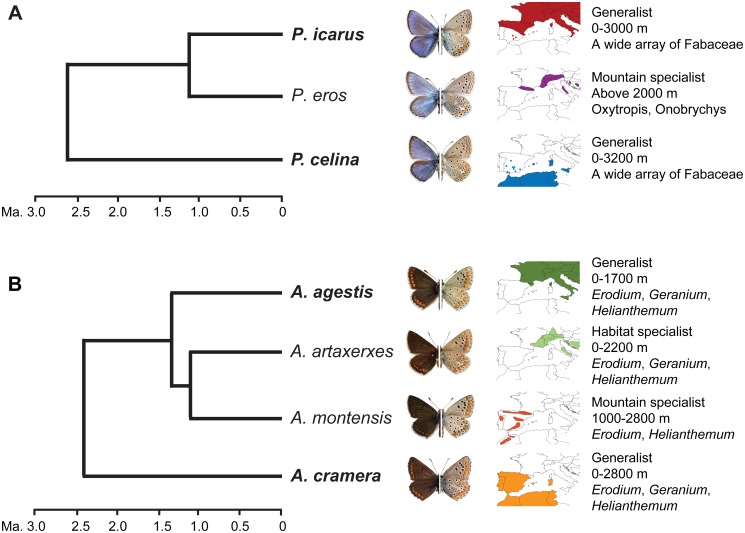
Model species. **A**. The evolutionary relationships, external appearance, western Mediterranean distribution and ecological preferences for *Polyommatus icarus*, *P*. *eros* and *P*. *celina*. *Polyommatus icarus* and *P*. *celina* are almost identical ecologically and morphologically, but *P*. *icarus* is phylogenetically closer to *P*. *eros*, a morphologically differentiated mountain species that feeds on a more restricted number of host plants. **B**. The evolutionary relationships, external appearance, western Mediterranean distribution and ecological preferences for *Aricia agestis*, *A*. *artaxerxes*, *A*. *montensis* and *A*. *cramera*. *Aricia agestis* is a habitat generalist phylogenetically the closest to a pair of specialist species:*Aricia artaxerxes* (boreo-montane) and *A*. *montensis* (montane). *Aricia cramera* is phylogenetically more distant to *A*. *agestis*, although both species are almost identical ecologically and morphologically.

Here, we indirectly test for the existence of mutual exclusion, using as a model two pairs of non-sister cryptic butterfly species: *Polyommatus icarus*-*P*. *celina*, and *Aricia agestis*-*A*. *cramera*. The genetic structure and evolutionary relationships of these species have been recently documented [21,22]. *Polyommatus icarus* and *P*. *celina* are parapatric, habitat generalist species, are found over a broad altitudinal range, and feed on a wide array of host plants (Fig. 1A). *Polyommatus celina* has been only recently recognized as a distinct species, and it can be reliably distinguished from *P*. *Icarus* based on both nuclear and mitochondrial genetic markers, while morphological traits only show minor trends of variation[21,28]. However, *P*. *icarus* is phylogenetically closer to *P*. *eros*, a genetically and morphologically differentiated mountain taxon that feeds on a more restricted number of host plants [21,28]. Similarly, *A*. *agestis* is a habitat generalist and ubiquitous Palaearctic species that is phylogenetically closer to the boreo-montane *A*. *artaxerxes* and the montane *A*. *montensis*. The sister of this clade is *A*. *cramera*, which occurs in the south-western Mediterranean and is almost identical to *A*. *agestis* in ecology and external morphology, but it is differentiated genetically and in the male genital morphology[22] (Fig. 1B). The recognition of these taxa as species or subspecies has been debated [29,30], but what it is important in our case is that the phylogenetically divergent pairs are parapatric and show higher morphological similarity than the sympatric and phylogenetic closer taxa. Moreover, the parapatric pairs are habitat generalists [31] that occur from sea level to high mountains, both in highly anthropic and in non-managed areas, and their varied host plants represent a virtually continuous and unlimited resource over space. Thus, it is unlikely that their distributions are constrained by habitat quality and host plant presence [10].

We sequenced a large number of specimens for all four cryptic species from the entire western Mediterranean region, with a special effort on the currently known contact areas (Iberia, the Sicily channel, the Messina channel and the strait of Bonifacio). We combined phylogeny, distribution modeling, genetic landscapes, and the evaluation of dispersal capabilities for each taxon and showed that the biogeographical patterns consistent with mutual exclusion for these cryptic species cannot be fully explained either by climatic preferences or by limited dispersal capabilities.

## Methods

### Ethics Statement

No specific permits were required for the studied areas in France, Italy and North Africa (Morocco, Algeria and Tunisia), because the locations were not privately owned or protected in any way, and the field studies did not involve endangered or protected species. All necessary permits for the field studies in Spain were obtained from the competent public bodies: environmental agencies of the Comunidades Autónomas, the Natural Parks of Sierra Nevada, Montseny and Picos de Europa, and Reserva Natural Illes Columbretes.

### Molecular data and genetic landscape

We analyzed the cytochrome *c* oxidase I(COI) mitochondrial marker for 325 *P*. *icarus*-*P*. *celina* and 262 *A*. *agestis*-*A*. *cramera* specimens from the western Mediterranean mainland and most of the islands in this region (S1 Table). Total genomic DNA was extracted using Chelex 100 resin, 100–200 mesh, sodium form (Biorad), under the following protocol: one leg was removed and introduced into 100 μL of Chelex 10% and 5 μL of Proteinase K (20 mg⁄ mL) were added. The samples were incubated overnight at 55°C and were subsequently incubated at 100°C for 15 minutes. Samples were then centrifuged for 10 seconds at 3.000 rpm. A 658-bp fragment at the 5’ end of the mitochondrial gene (COI) was amplified by polymerase chain reaction using the primers LepF1 (5´-ATTCAACCAATCATAAAGATATTGG-3´) and LepR1 (5´-TAAACTTCTGGATGTCCAAAAAATCA-3´) [32]. Double-stranded DNA was amplified through polymerase chain reactions (PCR) in 25-μL volumes containing: 14.4 μL autoclaved Milli-Q water, 5 μL 5x buffer, 2 μL 25 mM MgCl2, 0.5 μL 10 mM dNTPs, 0.5 μL of each primer (10 μM), 0.1 μL Taq DNA Polymerase (Promega, 5U/ μL) and 2 μL of extracted DNA. The typical thermal cycling profile was: first denaturation at 92°C for 60 s, followed by five cycles of 92°C for 15 s, 49°C for 45 s and 62°C for 150 s, and then by 35 cycles of 92°C for 15 s, 52°C for 45 s and 62°C for 150 s and a final extension at 62°C for 420 s. PCR products were purified and sequenced by Macrogen Inc. Sequences were edited and aligned using GENEIOUS PRO 6.0.5 created by Biomatters (http://www.geneious.com/). A part of the sequences generated by this study have been obtained at the Biodiversity Institute of Ontario, Canada. In this case a glass fibre protocol [33] was employed to extract DNA and PCR and DNA sequencing were carried out following standard DNA barcoding procedures for Lepidoptera [34]. All new sequences have been deposited in GenBank (accession numbers KM459029—KM459439, and KP052710). Neighbour-Joining (NJ) phylogenetic trees for identification purposes were obtained using MEGA 5.05 [35], with 100 bootstrap pseudo-replicates to assess the robustness of the tree clades.

To create genetic landscapes for each species pair, a matrix of p-distances and a table of GPS coordinates (decimal degrees format) for the corresponding samples were imported in R 3.0.2. When more than one sample belonged to the same location, mean genetic distances have been computed. Using the “deldir” package, we calculated a Delaunay triangulation among the GPS coordinates of the collection sites (S1 Fig.). Since the segments composing the triangles have different lengths and genetic distances tend to increase with geographic distance following different trends according to organisms and scale [36–38], we computed a series of preliminary regressions (linear with original values, linear with log-transformed values, and asymptotic regression) and selected the one showing the highest fit (R^2^). Subsequently, we computed residuals of the selected regressions between dissimilarities and geographical distances. For all pairs of areas connected in the Delaunay triangulations, we attributed the residual p-distance calculated between those areas to the midpoint of each segment [39]. The residual values and midpoint locations were imported in QGIS 2.0.1. (www.qgis.org), and the values interpolated using the inverse distance weighting algorithm to generate a visual representation of the spatial distribution of genetic divergence [39].

### Haplotype networks and evaluation of dispersal constraints

Haplotype networks for each species were inferred with the program TCS 1.21 from subsets consisting exclusively of sequences without ambiguities (S1 Table). On the basis of the relationships highlighted by these networks, it has been possible to identify a minimum number of sea crossing events necessary to produce the observed pattern of distributions. In practice, by assuming that genetic convergence is much less probable than dispersal, we scored a cross-sea dispersal event when a haplotype was shared between two areas separated by a sea strait. We also scored a dispersal event if two haplotypes occurring in areas separated by sea straits were directly linked in the haplotype network. When calculating the sea crossing capacity of the species, we used the-50m isobath, representing an approximate mean sea depth between glacial maxima and interglacial periods. Some of the investigated islands were connected to mainland areas according to this isobath (Elba, Pianosa and Ischia to Italy, Levant to France, Levanzo to Sicily) and hence no dispersal was scored in these cases. We conservatively excluded particularly long dispersal events when the genetic pattern could also be explained by extinction or non-detection of some haplotypes. As an additional source of information, we used island groups that are outside the studied area, but where some of the four studied species occur (eastern Mediterranean for *P*. *icarus* and *A*. *agestis*, Canary Islands for *P*. *celina* and *A*. *cramera*), and reconstructed the minimum number and shortest over-sea dispersal events required for these species to achieve the observed distributional pattern. Finally, we plotted the frequency of the observed dispersal lengths in order to verify if the unobserved events determining the chequered distributions lie within the inferred dispersal capability of each species.

### Ecological niche modeling

In order to test if the observed chequered distributions of the species pairs can be explained by climatic factors, we performed ecological niche modeling for each of the four species. Based on molecular results, reliable observations, and data from literature, we gathered over 7000 presence points for these species in the study area. For areas close to the contact zones we exclusively used presence points based on molecular results. In order to eliminate clustered occurrences due to unbalanced sampling, we filtered the datasets by randomly selecting points at a minimum distance of 0.5 degrees using the function gridSample of the R package “dismo”. The selection of 0.5 degrees has been made as a visual best compromise between maintaining the largest possible number of occurrence data and a good level of homogeneity. After filtering, we obtained 599 records for *P*. *icarus*, 128 for *P*. *celina*, 444 for *A*. *agestis* and 322 for *A*. *cramera* (S1 Dataset). We downloaded the 19 climatic layers from WorldClim (http://www.worldclim.org/, [5]) at a resolution of 30 arc-seconds. We cropped the layers to include only the western Mediterranean area and downscaled their resolution to 0.1 degrees of latitude and longitude to match the resolution of occurrence data. Climatic variables tend to be highly correlated mainly when regions with similar climate are analyzed. Collinearity does not largely affect the prediction of occurrence [40], but it may bias the estimate of the relative importance of predictors [6]. We evaluated the correlation among variables by randomly selecting 10,000 points of the WorldClim variables and analyzed the Pearson correlation between all pairs of variables. By inspecting the correlation matrix, we selected the maximum possible number of variables showing a Pearson correlation coefficient lower than 0.8 [41,42]. Eight out of 19 variables were retained: mean temperature diurnal range (°C), isothermality (°C), temperature seasonality (coefficient of variation in %), mean temperature of wettest quarter (°C), mean temperature of driest quarter (°C), precipitation of wettest period (mm), precipitation of driest quarter (mm) and precipitation of coldest quarter (mm). We then performed the ecological niche modeling analyses using the maximum entropy algorithm MaxEnt v3.3.3k. When using the Auto features, MaxEnt [6] generates five classes of predictor variables (linear, quadratic, product, threshold and hinge) often resulting in data over-fitting [43], and thus producing non-conservative results. According to recent reviews we performed two series of models. In the first series we only used hinge features (H models), which produce similar results to the generalized additive models (GAM)[44]. In the second series we used hinge, quadratic and product features (HQP models) because hinge features tend to be redundant with linear and threshold ones [44]. The distribution of each species was modeled using 100 replicates, subsample and random seed, with 25% of the presence data to test the model and 75% to train the model. All other settings were left by default. To plot the maps for the predicted species occurrences we considered as potentially suitable areas, those cells showing logistic values higher than the maximum training sensitivity plus specificity logistic threshold [45]. In order to test the importance of species interactions, we performed another set of species distribution modeling analyses in which we used the presence of the corresponding cryptic species as an additional categorical layer. The new layers were generated by alpha-convex hulls based on the presence data of each species. In order to obtain the best compromise between local convexity and the actual distribution of species we used the function alhull of the R package “alphahull” with the alpha value set to ten. Each 0.1 degree cell of the layers was categorized as 1 if internal to the convex hull (species occurring in the area of the cell) and as 0 if external (species not recorded to occur in the area of the cell). The convex hull layer of each species was then included as a categorical variable in a new MaxEnt analysis of its corresponding cryptic species.

In order to evaluate the relative importance of highly correlated predictors in the models, we inspected both the percent contribution of each variable to the model and the Jackknife output produced by MaxEnt. Because the Jackknife regularized training gain for models performed without one of the variables are expected uninformative in case of high collinearity, we used instead the training gain of the models, which tested the performance of each variable individually [41]. The least important variable returned by the climatic models was removed from the interaction analyses in order to use the same number of variables in both series of analyses.

## Results

### Locating hotspots of mutual exclusion: genetic landscape

**Fig 2:**
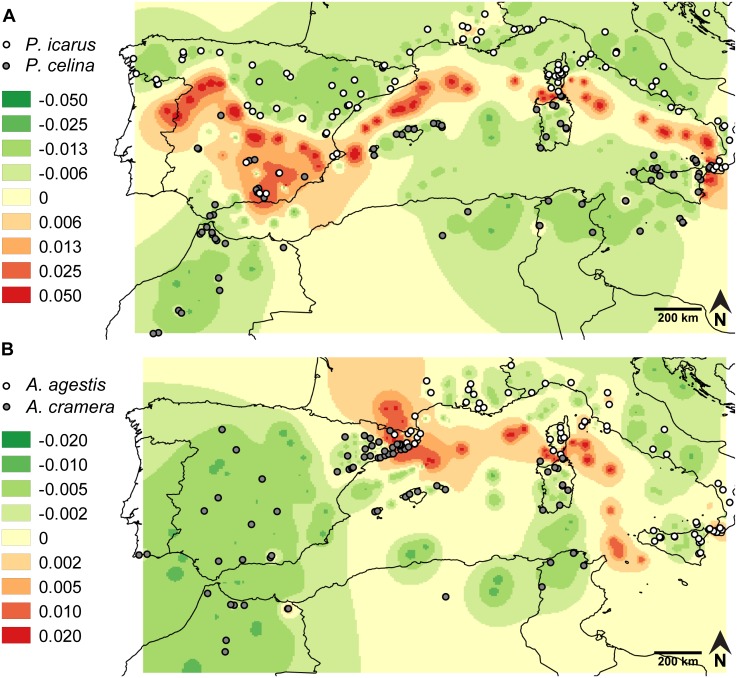
Genetic landscapes obtained for the sequenced specimens of the two pairs of cryptic species. The color gradient represents residuals of COI genetic p-distances. **A**. *Polyommatus icarus* and *P*. *celina*. **B**. *Aricia agestis* and *A*. *cramera*.

The phylogenetic trees based on COI sequences (S2 Fig.) resulted in clearly differentiated clades for each species in accordance with previous studies [21,22]. Among the regressions used to correct the genetic distances according to geographic distances, the relationship of original p-distances against log-transformed geographic distances showed the best fit. As expected for the reduced geographic scale involved in the Delaunay triangulation, the asymptotic regression failed in finding any significant solution. We thus computed and interpolated the residuals between p-distance and log-geographic distance. The resulting genetic divergence landscapes and the assignment of individuals to a species based on their position in the phylogenetic tree indicated, for both cryptic pairs, parapatry on mainland with a contact zone in the Iberian Peninsula, and not a single case of coexistence on islands (Fig. 2). For *Polyommatus*, the strongest genetic divergence with respect to geographic distance corresponded to abrupt changes of distributions across the narrow Messina and Bonifacio straits, along the Tyrrhenian Sea between Italy and Sardinia, between the Balearics and Iberia and between northern and southern Iberia. In southern Iberia the picture became more complex due to the existence of isolated populations of *P*. *Icarus* in particular mountaintops, such as Sierra Nevada and Sierra de La Sagra. For *Aricia*, the highest divergence emerged across the Bonifacio strait, along the Tyrrhenian Sea, between North Africa and Sicily, between the Balearics and France, in Catalonia and along the Pyrenees. As a result of intraspecific divergence, minor differentiation was also found between Corsica and the Tuscan Archipelago and between Sicily and the Italian Peninsula. In summary, for both species pairs the most pronounced genetic differences were located over sea areas, confirming that sea straits have strong power in the formation and maintenance of non-sympatric distributions.

### Evaluation of dispersal constraints based on haplotype networks

**Fig 3:**
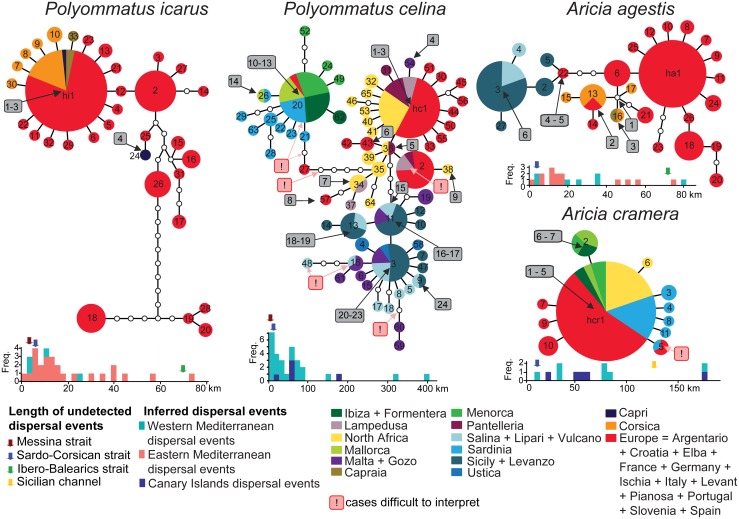
Haplotype networks and evaluation of dispersal events over the sea. Inferred over-sea dispersal events are numbered in grey rectangles, and ambiguous cases that were excluded from the analyses are highlighted in pink squares. The lengths of the dispersal events for the study area have been measured according to the-50m isobath and are displayed in histograms (cyan blocks), together with minimal dispersal events based on the distribution of the same species in the eastern Mediterranean (red blocks) and Canary Islands (blue blocks). The width of the key sea-straits Sardinia-Corsica, Italy-Sicily, Tunisia-Sicily and Ibero-Balearic are indicated by blue, red and green arrows, respectively.

Haplotype networks of the studied species showed different degrees of complexity, but in all cases intraspecific divergences were small enough so that the biogeographical history could be explained by Quaternary environmental changes and by the dispersal capabilities of the butterflies, instead of vicariance caused by older geological events. Regardless of the hypothetical location of the ancestor, the most parsimonious series of dispersal events that could have produced the observed patterns are highlighted in Fig. 3, S3 and S4 Figs. *Aricia cramera* showed a simple pattern with a widespread haplotype and some satellite ones connected by a single substitution (Fig. 3). Among dispersal events that could have produced the observed distributions (Fig. 3 and S3a Fig.), we retained that the pattern showed by haplotype hcr5, indicating dispersal from Spain to Sardinia, can be more parsimoniously explained by convergence or by the extinction or undetected presence of this haplotype in the Balearics. In order to obtain a conservative measurement of the frequencies of dispersal lengths, we excluded this ambiguous event. A minimum of five steps was required to attain the occurrence pattern of *A*. *cramera* over the Canary Islands (S3b Fig.). The dispersal length frequency, measured between the-50m isobaths, showed that the unrecorded Sardinia-Corsica dispersal event, potentially allowing *A*. *cramera* to mix with *A*. *agestis* populations in Corsica, would have been among the shortest dispersal events detected for this species. Conversely, the length of a hypothetical dispersal between Tunisia and Sicily is showed to be much less frequent (Fig. 3).

*Aricia agestis* showed a slightly more complex pattern. Corsica has been presumably colonized only once by an undetected haplotype that has drifted on both Corsica and mainland and successively reached Elba Island (connected to Italy in the-50 m isobath) and Capraia from Corsica (Fig. 3). Sicily has been apparently colonized in two events followed by differentiation and successive colonization of the Aeolian Islands. We thus scored six dispersal events (S3c Fig.), to which another between Sicily and Malta should be added because *A*. *agestis* was present in Malta at least until 30 years ago [46]. The examination of the distribution in the eastern Mediterranean, where only *A*. *agestis* is to be found, showed that a minimum of 14 dispersal events occurred in that region (S3d Fig.). Again, the Corsica-Sardinia dispersal would be among the shortest events recorded for this species, thus demonstrating that the dispersal capabilities have not been the limiting factor for the colonization of Sardinia by *A*. *agestis*.

According to previous results [21], *P*. *celina* showed three main lineages associated with i) Spain-Maghreb-southern Sicilian islands, ii) Balearics-Sardinia-Spain, iii) Sicily, circum-Sicilian islands. Spain and Malta harbour specimens belonging to two different lineages, which indicates that they can coexist in sympatry (S2 Fig.). According to such a complex pattern we scored a minimum of 24 overseas dispersal events (Fig. 3, S4a Fig.). We did not consider seven ambiguous events for the same reasons discussed for *A*. *cramera*. Four other events can be recognized for the Canary Islands (S4b Fig.). The comparison of the length of these events in the-50 m isobath with those required to colonize Italy from Sicily and Corsica from Sardinia revealed that such unrecorded dispersal events would have been among the shortest ones performed by this species.

Two well-separated lineages could be identified for *P*. *icarus*: one widely distributed over the whole Palaearctic region and one ranging from Sierra Nevada to France and Crete[21]. One Palaearctic haplotype found on Corsica, Capraia and Capri also occurs on mainland. Therefore, Corsica and Capraia seem to have been recently colonized and only once, while Capri has been colonized at least two times (Fig. 3). No evidence for other overseas dispersal events in the-50m isobath could be detected (Fig. 3, S4c Fig.). In the eastern Mediterranean at least 25 events(S4d Fig.) were necessary to justify the distribution on islands and most of them are longer than those required to reach Sicily from the Italian Peninsula and Sardinia from Corsica.

In summary, a number of successful colonization events were inferred from the haplotype networks and distributions on islands for the studied species. The lengths of the estimated sea-crossing dispersal events were frequently much longer than the distances required to cross the sea barriers where the most striking genetic divergences have been detected, especially for the narrow Messina and Bonifacio straits, just 3 and 5 kilometers long when considering the-50 m isobath (Fig. 3).

### Evaluation of climatic and interaction constraints: species distribution modeling

**Fig 4:**
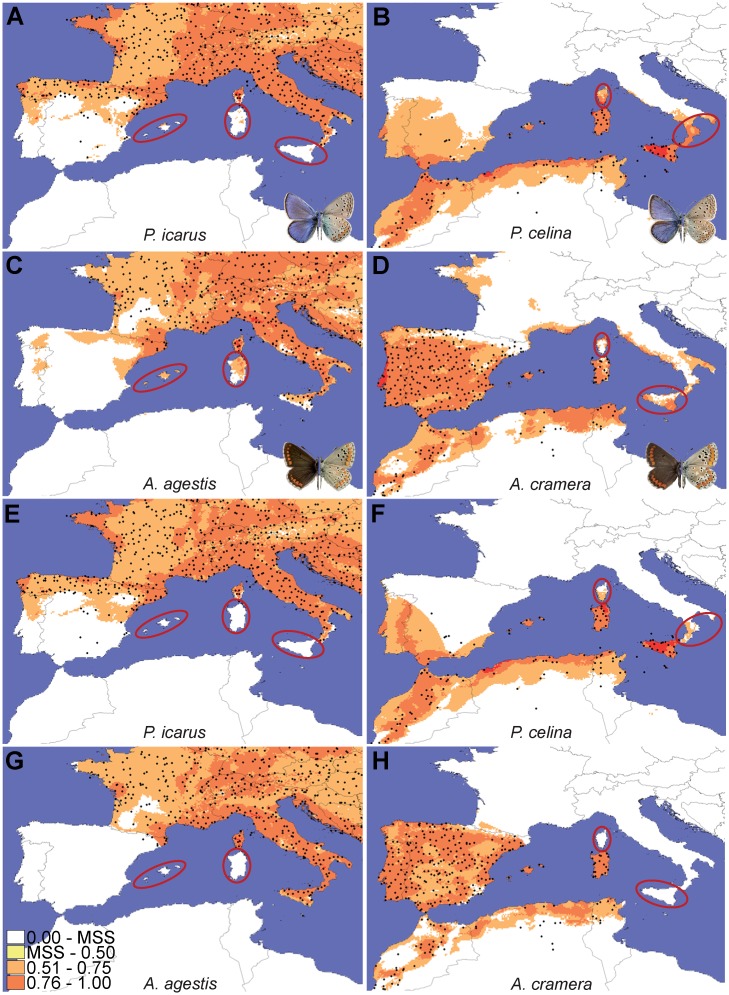
Projection over the study area of the logistic values obtained by MaxEnt analyses (H model) for the four species. The color gradient indicates the logistic probabilities of species occurrence, increasing from white (very low probability) to red (very high probability). The maximum value of the lowest class (white) is represented by the maximum training sensitivity plus specificity logistic threshold returned by MaxEnt. The key areas where the species are predicted to occur but are not present despite geographical proximity are highlighted.

**Fig 5:**
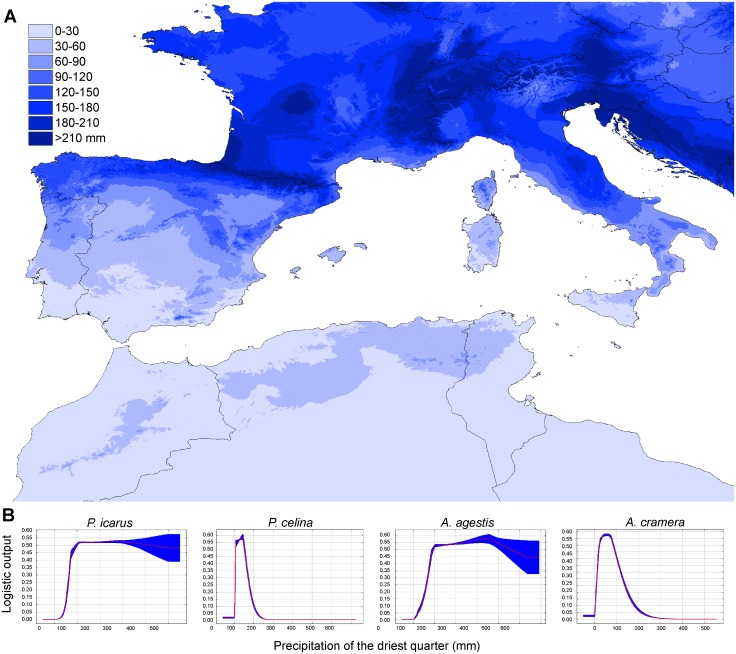
Projection of the climatic variable *precipitation in the driest quarter* and logistic responses for each species. **A**. The color gradient on the map indicates the precipitations (in mm) over the study area. **B**. The logistic responses of the precipitation in the driest quarter tested alone for the four studied species (*P*. *icarus*, *P*. *celina*, *A*. *agestis*, *A*. *cramera* from left to right). The response showed a clear and recurrent threshold between 50 and 100 mm of precipitation.

All the climatic models showed a good fit and with very similar results between H and HQP models (AUC for H models: *P*. *icarus* = 0.758; *P*. *celina* = 0.901; *A*. *agestis* = 0.791; *A*. *cramera* = 0.833; AUC for HQP models: *P*. *icarus* = 0.758; *P*. *celina* = 0.904; *A*. *agestis* = 0.792; *A*. *cramera* = 0.835; Fig. 4 and S5 Fig. for map projections). The climatic variable showing the highest percentage of contribution to the model in both the H and HQP analyses was precipitation of the driest quarter. This variable also showed the highest regularized gain when tested alone in the Jackknife evaluation for all species except *A*. *agestis*, for which the highest contribution was given by mean temperature diurnal range. By projecting the values of precipitation of the driest quarter on a map and comparing it with the plots representing the response of logistic occurrences of the four species (Fig. 5), it is clear that values of 50–100 mm of precipitation determine a threshold that is highly correlated with the distribution of the four species (Fig. 2).

According to the climatic models, only *P*. *icarus* was predicted to occur in areas that agreed with the actual distribution of the species, also between the two sides of the narrow sea straits of Messina and Bonifacio channels (Fig. 4A and S5a Fig.). For the other three species MaxEnt highlighted as climatically suitable large areas where they do not actually occur. These areas largely corresponded to the unoccupied sides of the sea straits identified as areas with the strongest genetic contrasts (Sicily-Calabria, Sardinia-Corsica, North Africa-Sicily, Iberia-Balearics; compare Fig. 2 and Fig. 4A–D and S5a–d Fig.), thus indicating that climatic constraints cannot entirely explain the chequered distributions of three of the studied species.

The second series of maximum entropy models, in which we included the convex hull representing the presence area of the corresponding cryptic species as a categorical predictive layer, revealed a strong influence of the presence of the peer cryptic species in determining the observed pattern. The models revealed high AUC values also in this case (AUC for H models: *P*. *icarus* = 0.754; *P*. *celina* = 0.902; *A*. *agestis* = 0.799; *A*. *cramera* = 0.867); AUC for HQP models: *P*. *icarus* = 0.755; *P*. *celina* = 0.904; *A*. *agestis* = 0.805; *A*. *cramera* = 0.904). The presence of the related cryptic species was indicated as the variable showing the highest percentage contribution to the model for all species except *P*. *icarus*, for which it is ranked as second after precipitation of the driest quarter. Conversely, in the Jackknife evaluation of the regularized gain, the presence of the related cryptic species was the most important variable only for *A*. *cramera*. In these analyses, logistic predictions for presence/absence along sea straits showed a much higher correspondence with the observed patterns than those obtained only with climatic variables (Fig. 4E–H and S5e–h Fig.).

## Discussion

This study shows that the strongest genetic contrasts between the two pairs of cryptic species occur over short distances, which prominently correspond to the Bonifacio and Messina straits. On the basis of the estimated dispersal capabilities, we show that each species are able to cross much longer sea barriers than Bonifacio and Messina. The two pairs of cryptic species revealed strong climatic preferences but these preferences alone could not fully explain the observed distribution pattern. In fact, on the basis of distribution modeling, both cryptic pairs were expected to coexist at least on one side of these relatively narrow sea straits. Conversely, only one of the two sibling species was documented on each side (Figs. 3 and 4), although dozens of individuals from the potential contact zones were examined (S1 Table). Previous papers suggested potentially chequered distribution patterns for several butterfly taxa in the Mediterranean region [20–22,47,48] and a recent study showed that the cryptic butterfly diversity in this region is overwhelmingly composed by groups of species that are not sympatric [19]. Since geographic isolation is considered to be the main driver for speciation, the tendency for sister species to show allopatric distributions is to be expected. However, the complete segregation of species and lineages over narrow sea straits that we observed is intriguing. Several hypotheses can be proposed to explain this pattern.

### Dispersal capabilities and ecological constraints

A direct comparison of dispersal capabilities against the observed barriers potentially maintaining the vicariance patterns failed to explain the observed distribution. This is not surprising since recent studies have shown that butterflies can experience range expansions/contractions even over relatively short periods of time [20,49–51] and can rapidly adjust their distribution to track suitable environments [49]. The absence of specific resources, such as host plants, is unlikely to be an explanation for the observed mutual exclusion, because the four studied species are habitat and trophic generalists, occurring in a wide array of environments, from anthropic to mountain areas, and feeding on a variety of similar, and even identical, ubiquitous plant resources [52–54]. Since the host plants for the more recently discovered species *P*. *celina* were not well documented, we provide a table of our field observations (S2 Table) showing that it feeds on at least four widespread genera of Fabaceae. Species distribution modeling revealed that the two pairs of species experience different climate settings in the areas where they occur and that a common threshold of about 50–100 mm of precipitation in the driest quarter (summer in the Mediterranean) is highly correlated with all the observed distributions. Warm and dry conditions in the Mediterranean are well known to affect the life history of many butterfly species which, in many cases, emerge at the beginning of summer and estivate to delay reproduction in colder and wetter conditions [55]. There is growing evidence that interspecific variation in mitochondrial genes can determine a different respiration metabolism [56]. The strong differences in COI among the morphologically similar studied species may reveal to be functional for surviving in different climate settings and to be directly involved in maintaining the observed chequered patterns. However, it is difficult to evaluate if the observed climatic threshold has a real causal effect in determining the spatial separation among the species or if it only coincides with three main areas of phylogeographic breaks (Iberia-Maghreb, Sardinia-Corsica, Maghreb-Sicily-Calabria). Nevertheless, when included in the model, the presence of the corresponding cryptic pair had a strong influence in the models and explained most of the observed discordances between climatic predictions and the actual distribution of the four species. This suggests that the examined cryptic pairs have been influenced by the presence of other members of the same group in the recipient areas, apparently as much as by temporal and physical constraints.

### Competition

Studies focused on birds demonstrated a correlation between differences in functional beak morphology and the rate of secondary sympatry, which suggests that competition instead of sexual interference was the main determinant of chequered distributions [17,18]. However, we specifically selected two pairs of species with high genetic divergence, thus with long potential time for secondary contacts, and a high degree of generalism, which renders competition for resources rather unlikely [10]. The evidence for a highly nested structure in butterfly communities, assembled in an order that reflects well their degree of specialization, provides empirical evidence that generalist butterflies do not tend to exclude each other [57]. Indeed, most of the widespread species of butterflies are habitat generalists that have the tendency to largely co-occur [58]. Thus, the two pairs of species studied here show idiosyncratic distributions beyond the general hypothesis stating that sister species are primarily allopatric and maintained their distribution pattern due to a short dispersal and evolutionary time, but competition for resources does not seem to be a suitable alternative explanation either.

### Reproductive interference

It should be noted that the observation of strict mutual exclusion among these pairs of species only applies to islands, because on restricted mainland areas these cryptic species are known to display contact zones [21,22] where occasional potential hybrids are found. Accordingly, distribution modeling showed that the two pairs experience different climatic conditions but also that suitable areas largely overlap. Presumably, no strong precopulatory barriers exist between these species, as is the case in many butterflies [59,60], but hybrids between closely related species often display reduced fitness [30,61]. In the absence of precise recognition mechanisms, contact areas can be seen as population sinks, unlikely to enlarge given the cost for the neighboring populations [26]. Moreover, hybrid zones are predicted to shift until areas allowing low dispersal and low population densities are reached [26,62]. A concentration of boundaries between cryptic species over sea straits located over areas separating the climatic preferences of the different taxa, highly matches this hypothesis [19]. Thus, we hypothesize that the observed mutual exclusion, at least for the two pairs of cryptic species studied here, could be mainly due to a combination of climatic preference reinforced by reproductive interference between species [63].

Interestingly, our data on *P*. *celina* show that different lineages coexist in Malta (Sicilian and North African lineages) and Spain (European and Sardo-Corsican lineages), suggesting that multiple successful colonization events over notably long distances can take place, but only when propagules involve the same species as recipient populations (a case in which reproductive interference would not exist). In fact, these observations suggest that the niche carrying capacities in these islands are not saturated and allow the establishment of additional incoming lineages.

## Conclusion

There is comparative evidence in literature that interactions between sister species constrain their range expansion [17,19]. Based on a completely different approach, our results point to the conclusion that a combination of climatic preferences and biotic interactions limit geographic range overlap and reject models limited purely by dispersal constraints. We show that this phenomenon can be more acute in particular non-sister cryptic than in non-cryptic sister taxa, which agrees with the positive correlation found between morphological distance and secondary contact [17], but not with that of phylogenetic distance versus secondary contact, since phylogenetically nearer species (*P*. *icarus-P*. *eros* and *A*. *agestis-A*.*artaxerxes/montensis*) are largely sympatric. Our data support productive interference plus climatic preference hypothesis over ecological competition, but the mechanisms contributing to the realized distributions may vary depending on the taxonomic group.

Climatic preference and density-dependent processes have been supposed to be key factors in determining the evolution of species, their mutual exclusion on oceanic islands and in generating and maintaining the phylogeographic structures of many species in Europe [21,22,47,48]. Indeed, density-dependent processes, mostly at the leading edge of colonization events, can generate striking geographic contrasts in the distribution of genes and species. We hypothesize that a well-established population of a taxon in a recipient area can strongly interfere with the dispersing individuals belonging to the other cryptic taxon, and thus maintain the geographic patterns established over sea straits by a “founder takes all” mechanism [64]. With increasing knowledge of cryptic Mediterranean butterflies there is evidence for recurrent separation of different species/lineages over the Bonifacio and Messina channels (e.g. *Spialia orbifer/sertorius*, *Melanargia arge/pherusa* [65], *Coenonympha pamphilus/lyllus* [66], *Lysandra coridon* group [67], *Pararge aegeria* lineages [20]). This pattern is reinforced by the observation that among all western Mediterranean butterfly species, cryptic taxa tend to establish contact zones over the same sea straits [19]. Notably, the Messina and Bonifacio straits, but also the area between Iberia and Maghreb, show a similar abrupt change in the quantity of precipitations during the driest quarter. Precipitation is an important factor influencing butterfly survival in the Mediterranean, since it determines availability of key resources for adults and larvae (water, nectar sources, host plants)[55]. Thus, the phenomenon studied here for two pairs of species may have a prominent impact in determining the overall patterns of butterfly diversity in the region.

The mechanisms here discussed are theoretically applicable to most organisms, and a direct implication of our results is the necessity to consider interactions among cryptic entities when aiming at documenting diversity and its dynamics, including the effects of global changes [49,68,69]. Unfortunately, including the cryptic fraction of biodiversity is not straightforward because its complex recognition requires extensive morphological and/or genetic assessments, and these species are frequently amalgamated in wide-scale surveys [30,49,70].

## Supporting Information

S1 Dataset GPS coordinates for the specimens used in the ecological niche modeling. (XLSX)

S1 Fig Delaunay triangulation obtained for the sequenced specimens of *Polyommatus* spp. and *Aricia* spp. (TIF)

S2 Fig Neighbour-Joining trees based on COI sequences for *Polyommatus* spp. and for *Aricia* spp. (TIF)

S3 Fig Minimum dispersal events recorded for *Aricia* spp. (TIF)

S4 Fig Minimum dispersal events recorded for *Polyommatus* spp. (TIF)

S5 Fig Projection over the study area of the logistic values obtained by MaxEnt analyses for the four species, with the HQP models. (TIF)

S1 Table List of specimens used in the molecular analysis. (XLSX)

S2 Table List of host plant records for *Polyommatus celina*. (XLSX)
