# Supplementary figures and images for: Why Do Cryptic Species Tend Not to Co-Occur? A Case Study on Two Cryptic Pairs of Butterflies
Source: PLoS One. 2015 Feb 18;10(2):e0117802. doi: 10.1371/journal.pone.0117802 (PMC4334660; doi:10.1371/journal.pone.0117802)

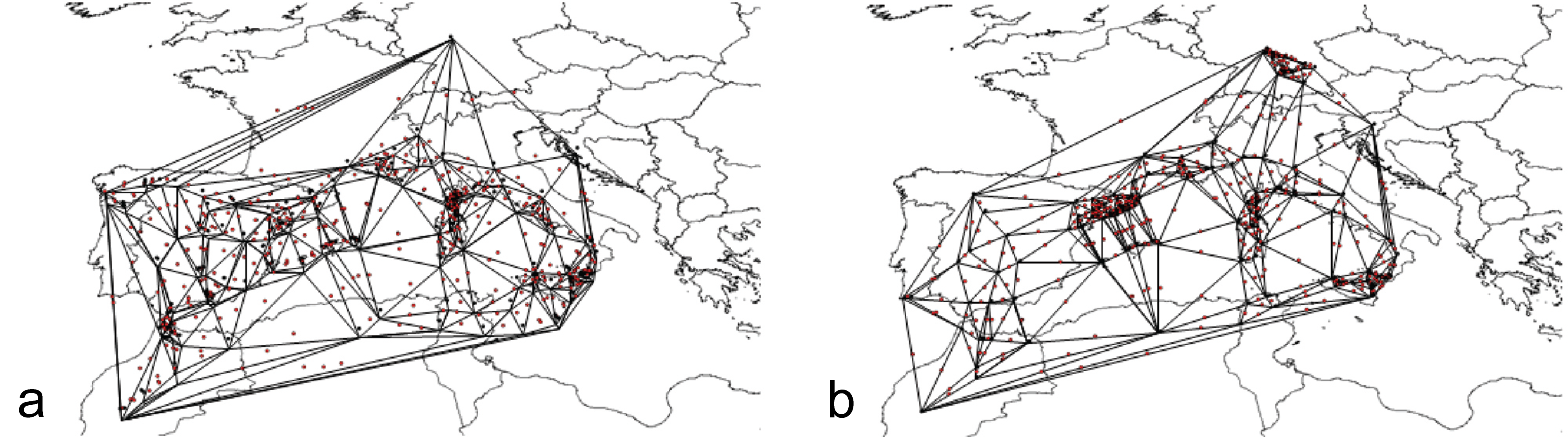

Supplement: S1 Fig — (TIF) [file pone.0117802.s002.tif]

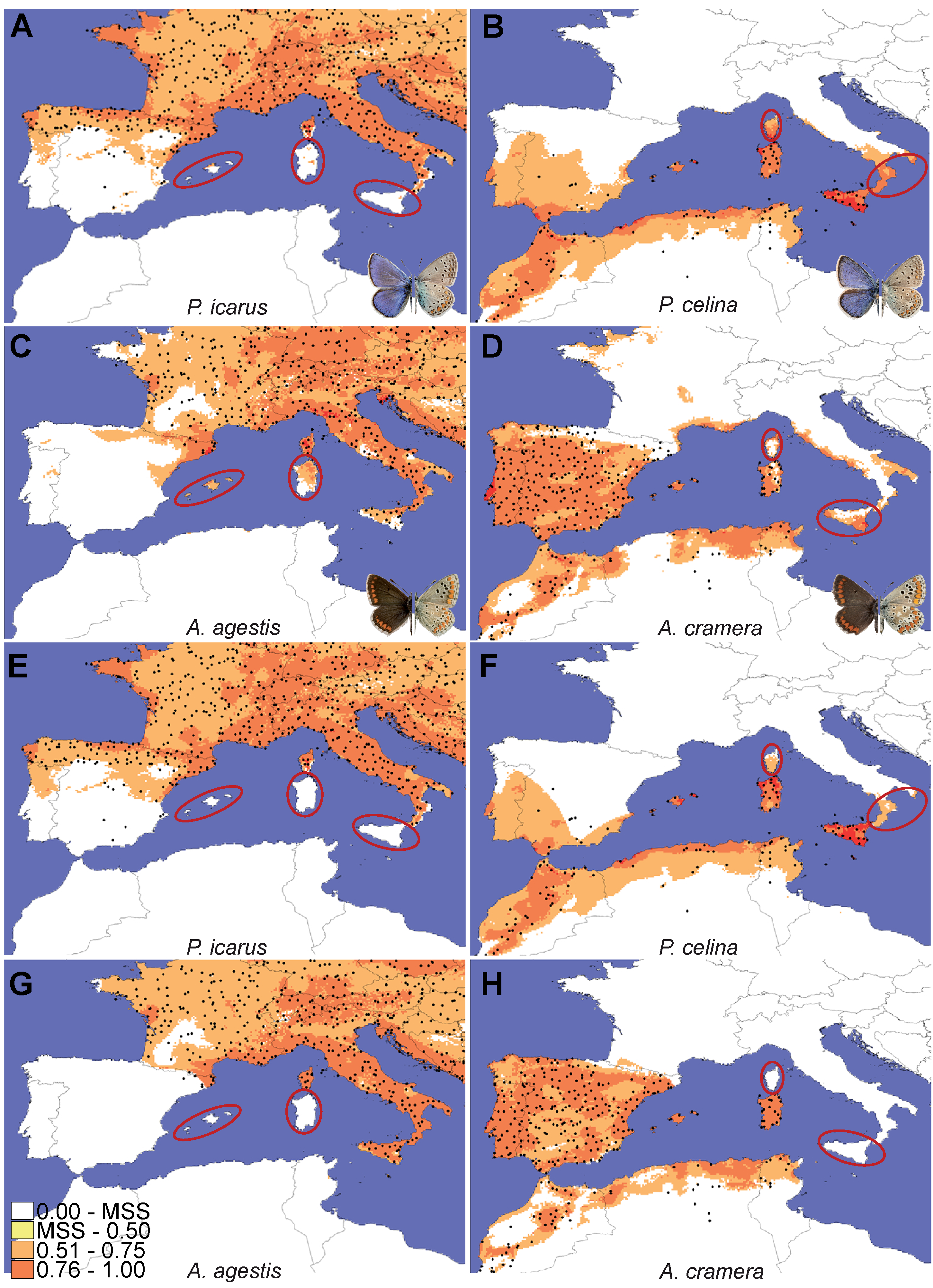

Supplement: S5 Fig — (TIF) [file pone.0117802.s006.tif]
